# Assessment of Patient Expectations and Preferences Following OPHL: Results from an Evaluation Study

**DOI:** 10.3390/curroncol33010063

**Published:** 2026-01-21

**Authors:** Erika Crosetti, Francesca Piccinini, Anastasia Dyrda, Daniela Nassisi, Marco Fantini, Giovanni Succo

**Affiliations:** 1 ENT Department, Head Neck Cancer Unit, San Giovanni Bosco Hospital, 10154 Turin, Italy; francesca.piccinini@aslcittaditorino.it (F.P.); giovanni.succo@unito.it (G.S.); 2 Oncology Department, University of Turin, 10124 Turin, Italy; anastasia.dyrda@edu.unito.it; 3 Radiotherapy Service, Mauriziano Umberto I Hospital, 10128 Turin, Italy; dnassisi@mauriziano.it; 4 ENT Unit, Koelliker Hospital, 10134 Turin, Italy; marcofantini8811@hotmail.it; 5 ENT Unit, San Feliciano Hospital, 00143 Rome, Italy

**Keywords:** OPHL, laryngeal partial surgery, patient expectation, laryngeal cancer

## Abstract

Open partial horizontal laryngectomy (OPHL) is a key conservative option for laryngeal cancer, with established oncological outcomes but limited data on functional results and patient perspectives. Voice preservation is mainly associated with type I OPHL, whereas types II–III often result in significant but broadly comparable impairments, making vocal decline the main limitation of OPHL. Patient-reported outcomes (PROs) help clarify the balance between treatment efficacy and side effects. This single-institution study analyzed 70 consecutive OPHL patients (12 women, 17.1%; 58 men, 82.9%), mean age 65.9 years (SD 8.96), with a median follow-up of 52.5 months (range 2–218). PROs were assessed using the Priority Scale, the V-RQOL, the MDADI, the Decisional Conflict Scale, the Decisional Regret Scale, and the Brief Pain Inventory. The Priority Scale showed that curing cancer (98.6%) and prolonging life (82.9%) were top concerns, while only 34.3% prioritized natural voice preservation. V-RQOL averaged 77.4/100, indicating limited impact of voice on quality of life; MDADI was 78.5/100, reflecting minimal swallowing difficulties. Decisional Conflict averaged 34.3/100, with 30% reporting no difficulty; Decisional Regret was low (13.0/100), with only 1.4% expressing moderate regret. Most patients (78.6%) reported no pain. Overall, OPHL provided satisfactory functional and decisional outcomes, with high patient satisfaction despite the complexity of treatment.

## Simple Summary

Laryngeal cancer can affect breathing, speech and swallowing, and treatment often requires balancing disease control with the preservation of daily functions such as voice. Open partial horizontal laryngectomy (OPHL) is a surgical procedure designed to remove the tumor while preserving part of the larynx, but its impact on patients’ quality of life is not fully understood. This study aimed to explore what matters most to patients before surgery and how satisfied they feel afterward. We analyzed the experiences of 70 people treated with OPHL and asked them to report their priorities, their voice and swallowing abilities, their level of pain, and whether they had doubts or regrets about their decision. Most patients said that curing the cancer and living longer were their main goals, while keeping a natural voice was less important. After surgery, most reported good quality of life regarding both voice and swallowing, very low levels of regret, and little or no pain. Only a small number of patients experienced substantial uncertainty regarding their treatment choice. Overall, these results suggest that OPHL offers an acceptable balance between cancer control and everyday functioning, and may provide valuable information to patients, families, and caregivers when discussing treatment options.

## 1. Introduction

Open partial horizontal laryngectomies (OPHLs) represent a valuable therapeutic option for patients with intermediate- to advanced-stage laryngeal squamous cell carcinoma (LSCC). They offer an organ-preserving alternative to total laryngectomy (TL) or chemoradiotherapy (CRT), with the aim of maintaining essential laryngeal functions—phonation, swallowing and breathing—while achieving comparable oncological outcomes. Moreover, continuous improvements in surgical technique have minimized functional impairments, enhancing postoperative recovery and contributing to better overall patient health [1,2].

Health-related quality of life (QoL) in patients undergoing partial laryngectomy has been extensively studied using a variety of validated instruments. Among the most widely used are the European Organization for Research and Treatment of Cancer Core Quality of Life Questionnaire (EORTC QLQ-C30) and its head and neck cancer-specific module (EORTC QLQ-H&N35), which assess physical, social and psychological domains of well-being [2,3]. Additional tools include the Voice-Related Quality of Life (V-RQOL) questionnaire, which measures the impact of vocal changes on daily functioning [4,5], and the SF-36, a general health survey often used to compare cancer patients with the general population [4,6].

These tools enable clinicians to monitor a wide range of functional and psychosocial aspects, including communication ability, emotional well-being, and difficulties related to swallowing and respiration [1,3,7]. Overall, patients treated with OPHLs generally report a better quality of life than those who undergo total laryngectomy, particularly with regard to communication ability and preservation of swallowing function [3,5,8]. However, considerable individual variability in post-treatment adaptation underscores the importance of addressing not only physical but also psychological dimensions of recovery.

The psychological aspect of quality of life is often underestimated, yet it plays a pivotal role in the post-operative rehabilitation process. Patients with a higher predisposition to chronic worry or negative emotional coping tend to report worse outcomes, whereas those who succeed in maintaining social integration and adopting positive coping strategies exhibit more favorable long-term results [6,9]. This evidence supports the need for a comprehensive, multidisciplinary approach that includes not only oncological and functional follow-up, but also psychological support tailored to help patients accept their altered physical condition and enhance overall satisfaction with surgical outcomes [2,5,8].

In this context, there is a growing need to further explore the psychological dynamics involved in recovery following partial laryngectomy. Quality of life should not be considered solely in terms of regained physical function, but also through the lens of the patient’s own perceptions, emotional responses and ability to resume social and professional life [2,5]. Greater emphasis on psychological well-being and subjective satisfaction could foster more effective and individualized rehabilitation strategies [3,10].

The primary goal of the present study was to identify the priorities that patients subjected to OPHLs consider most relevant to their well-being prior to surgery. Based on these priorities, a structured evaluation was conducted postoperatively using specific measurement tools to assess whether patients’ preoperative expectations had been met. In addition, the study explored feelings of uncertainty and regret associated with the decision to undergo surgery, aiming to gain a comprehensive understanding of patients’ psychophysical status. A secondary objective was to evaluate the overall level of postoperative satisfaction and the possible presence of decisional regret following surgery.

## 2. Materials and Methods

This study was conducted on a cohort of 71 patients, including 58 males (81.69%) and 13 females (18.31%), with a mean age of 65.5 ± 9.42 years, all of whom underwent open partial horizontal laryngectomy (OPHL) for laryngeal carcinoma between 2005 and 2024.

All patients underwent the same clinical assessment during the three weeks prior to surgery, including clinical examination, nutritional status evaluation, videoendoscopy (laryngostroboscopy and NBI endoscopy), neck MRI/CT, histopathological confirmation by biopsy, and discussion within a multidisciplinary tumor board (MDT).

All patients were enrolled in a rigorous clinical and radiological follow-up program, consisting of quarterly clinical and endoscopic evaluations and biannual imaging assessments.

Demographic characteristics of the study population are summarized in Table 1. Following informed consent, all patients were administered six validated questionnaires during routine follow-up visits. In cases where in-person evaluation was not feasible, the questionnaires were administered through structured telephone interviews. Artificial intelligence assistance was used for English translation and grammatical refinement.

### 2.1. Assessment Instruments

This study focused on the outcomes derived from a battery of validated questionnaires, including the Priority Scale, the Voice-Related Quality of Life (V-RQOL) Measure, the MD Anderson Dysphagia Inventory (MDADI), the Decisional Conflict Scale (DCS), the Decisional Regret Scale (DRS), and the Brief Pain Inventory (BPI).

### 2.2. Priority Scale

The Priority Scale, introduced by Sharp et al. in 1998 [12], is a tool developed to identify the treatment-related priorities of head and neck cancer patients. It supports shared decision-making by clarifying patient preferences when choosing between treatments with different side effects, such as surgery versus radiotherapy.

Based on cognitive interviews with long-term survivors and a literature review, the scale includes 12 treatment-related outcomes considered most important by patients. Participants are asked to group these outcomes into three levels of priority (high, medium, low) and then rank them within each group, resulting in an overall order from 1 to 12.

Analysis focuses on how often each item appears among the top four priorities, highlighting both individual and group trends. Patient preferences often change over time—for example, favoring voice or swallowing function before treatment, and pain control or survival afterward.

The Priority Scale helps tailor treatment plans to what patients value most, ensuring a more patient-centered approach.

### 2.3. Voice-Related Quality of Life (V-RQOL)

The Voice-Related Quality of Life (V-RQOL) questionnaire, developed by Hogikyan et al. in 1999 [13], assesses the impact of voice disorders on daily functioning and emotional well-being. Initially composed of 12 items, it was later reduced to 10 for improved reliability and ease of use.

The items cover two domains: physical functioning (e.g., speaking in noisy environments, phone use) and socio-emotional functioning (e.g., anxiety, social withdrawal). Responses are rated on a 5-point Likert scale (1 = no problem; 5 = worst possible problem). Scores are calculated for each domain and as a total, normalized to a 0–100 scale, with higher scores reflecting better quality of life.

The V-RQOL is a valid, reliable, and sensitive tool that offers a patient-centered view, complementing objective voice assessments and supporting clinical decision-making.

### 2.4. MD Anderson Dysphagia Inventory (MDADI)

The MD Anderson Dysphagia Inventory (MDADI), developed by Chen et al. in 2001 [14] and validated in Italian by Schindler et al. in 2008 [15], assesses the impact of dysphagia on quality of life in head and neck cancer patients.

It includes 20 items across three domains: functional (daily activities), emotional (psychological impact), and physical (symptoms and severity). Responses are rated on a 5-point Likert scale (“Strongly disagree” to “Strongly agree”).

Scores are combined and normalized on a 0–100 scale, with higher scores indicating better quality of life. The MDADI is a reliable, patient-centered tool for evaluating both functional and psychosocial effects of dysphagia in clinical and research contexts.

### 2.5. Decisional Conflict Scale (DCS)

The Decisional Conflict Scale (DCS), developed by O’Connor et al. in 1995 [16], measures patients’ uncertainty and difficulty during health-related decision-making.

It includes three subdomains: decisional uncertainty, contributing factors (e.g., lack of information, unclear values, external pressure), and perceived decision quality (clarity, alignment with values, and feasibility).

Participants rate statements on a 5-point Likert scale (“Strongly agree” to “Strongly disagree”) based on a current or recent decision. Total and subdomain scores are calculated, with higher scores indicating greater decisional conflict.

The DCS supports informed decision-making and helps tailor individualized interventions in clinical settings.

### 2.6. Decisional Regret Scale (DRS)

Closely related to the DCS, the Decisional Regret Scale (DRS) was developed by Brehaut et al. in 2003 [17] to measure emotional distress or regret following medical decisions, particularly treatment choices. It offers a standardized, valid, and reliable tool to quantify decision-related regret—an emotion that can affect patient satisfaction and future behavior, especially in value-sensitive decisions where no single clinical option is clearly superior.

The DRS consists of five statements assessing different aspects of regret, such as whether the decision was right, whether the patient would make the same choice again, and whether the decision caused harm.

Responses are rated on a 5-point Likert scale (1 = “Strongly agree,” 5 = “Strongly disagree”). The average score is converted to a 0–100 scale, with higher scores indicating greater decisional regret.

The DRS helps identify patients dissatisfied with their choices and provides insights into how they reflect on decisions over time. Results can guide strategies to improve shared decision-making and reduce emotional distress.

### 2.7. Brief Pain Inventory (BPI)

The Brief Pain Inventory (BPI), developed by Daut and Cleeland in 1983 [18] and validated in Italian by Bonezzi in 2002 [19], is a tool used to assess pain and its impact on quality of life. Initially created for cancer patients, it has since been adapted for evaluating chronic non-cancer pain in various clinical settings.

The BPI measures two main aspects: pain intensity and its interference with daily life, including mood, sleep, physical activity, and social interactions. It consists of two sections: (1) a pain map, where patients mark painful areas on a body diagram; (2) numeric rating scales (0–10) to assess pain severity and interference, with 0 indicating no pain and 10 the worst imaginable pain.

Patients also report the percentage of pain relief achieved with treatments and the extent to which pain interferes with seven life domains: work, recreational activities, walking, sleep, mood, relationships, and overall quality of life.

## 3. Results

A total of 71 patients were enrolled in the study. The mean age at the time of analysis was 65.8 years (SD ±9.42), while the mean age at surgery was 60.7 years (SD ±8.74). The majority of patients were male (81.69%), and most were active smokers (56 patients, 78.87%), with 7 patients (9.86%) reporting regular alcohol consumption. A subset of 13 patients (18.31%) had previously undergone transoral CO_2_ laser surgery, including type I cordectomy in 2 cases (15.38%), type II in 4 (30.77%), type III in 2 (15.38%), type IV in 1 (7.69%), and type V in 4 (30.77%).

Histopathological examination revealed squamous cell carcinoma in 67 cases (94.37%), chondrosarcoma in 3 cases (4.23%), and papillary thyroid carcinoma in 1 case (1.41%). Clinical and pathological staging was performed according to the 8th edition of the TNM classification system. Surgical procedures, including the type of open partial horizontal laryngectomy (OPHL) and neck dissection, are summarized in Table 1.

Adjuvant therapy was indicated in 14 patients (19.72%) based on the presence of high-risk pathological features, including positive surgical margins, extralaryngeal extension (pT4a), and nodal metastasis. Of these, 7 patients (9.86%) received radiotherapy, 6 (8.45%) underwent chemoradiotherapy, and 1 (1.41%) was treated with radioiodine therapy.

The mean follow-up duration was 52.4 months (SD ±46.77), ranging from 12 to 240 months. During follow-up, disease recurrence occurred in 4 patients: 2 (2.82%) with regional relapse, 1 (1.41%) with local and regional recurrence, and 1 (1.41%) with distant metastases. Salvage surgery followed by chemoradiotherapy was performed in 3 cases (75%), while 1 patient (25%) was treated with immunotherapy. The calculated disease-free survival (DFS) rate was 93.6%. At the most recent follow-up, 70 patients (98.59%) were alive and disease-free, while 1 patient (1.41%) had persistent distant disease.

### 3.1. Priority Scale Results

Among the Priority Scale responses (Table 2), the items most frequently ranked among the top three priorities were: “being cured of my cancer” (70 times, 98.6%), “living as long as possible” (59 times, 83.1%), “maintaining my natural voice” (24 times, 33.8%), and “being free of pain” (20 times, 28.2%).

In contrast, the least prioritized items—most often ranked among the bottom three—were: “returning to my usual activities as soon as possible” (37 times, 52.1%), “maintaining my physical appearance” (43 times, 62.0%), and “having a comfortable level of mouth moisture” (48 times, 67.6%).

An age-stratified analysis compared responses between younger (≤65 years) and older (>65 years) patients. Older patients placed a significantly greater importance on “returning to usual activities as soon as possible” (*p* = 0.05). Conversely, younger patients tended to prioritize “maintaining physical appearance”, though the difference was not statistically significant (*p* = 0.07) (Table 2).

**Figure 1:**
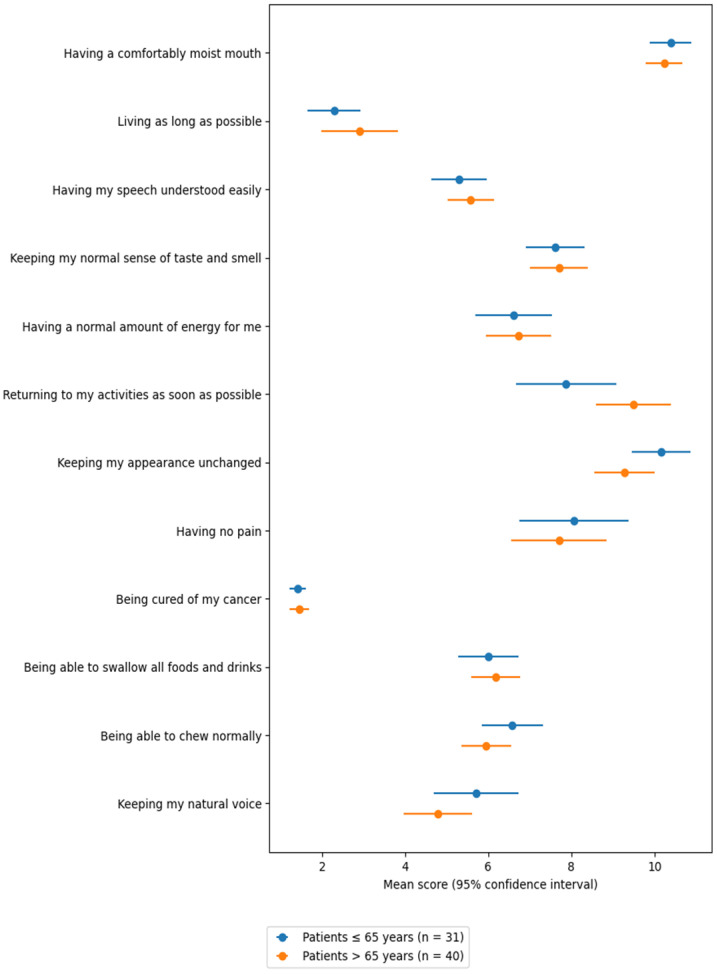
Dot plot showing mean scores with 95% confidence intervals for patient-reported outcome priorities, stratified by age group (patients ≤ 65 years, *n* = 31; patients > 65 years, n = 40). For each item, dots represent mean values and horizontal bars indicate 95% confidence intervals calculated from the reported standard deviations and sample sizes.

In univariate analysis, age > 65 was associated with a statistically significant mean increase of 1.62 points in priority ranking for the item “returning to usual activities as soon as possible” (*p* = 0.034; adjusted R^2^ = 0.05) (Figure 1).

Since questionnaires were collected at variable time points after surgery—ranging from a few months to nearly 20 years—this factor could potentially influence the results, particularly with regard to response reliability and recall bias. To address this concern, an additional stratified analysis was performed according to postoperative interval. The cohort was divided into two groups (patients operated between 2005–2015 and those operated from 2016–2024), and the Priority Scale rankings were recalculated within each subgroup. The findings were consistent across both strata: (1) in both groups, the first priority remained “being cured of my tumor”; (2) the second priority was “living as long as possible”; (3) the third priority was “maintaining my natural voice,” although in the 2005–2015 group this was tied with “being free of pain”; and (4) in the 2016–2024 group, “being free of pain” ranked fourth. These stratified results indicate that the relative ranking of patient priorities remains stable, even when considering variability in postoperative timing, thereby supporting the robustness of the findings.

### 3.2. Voice-Related Quality of Life (V-RQOL)

The mean V-RQOL score among patients was 77.4 out of 100 (SD ± 12.85), indicating a generally preserved voice-related quality of life. A detailed distribution of patients by score category (poor, fair, good, very good, excellent) is presented in Table 3. Higher scores reflect a lower negative impact of voice on overall quality of life.

**Figure 2:**
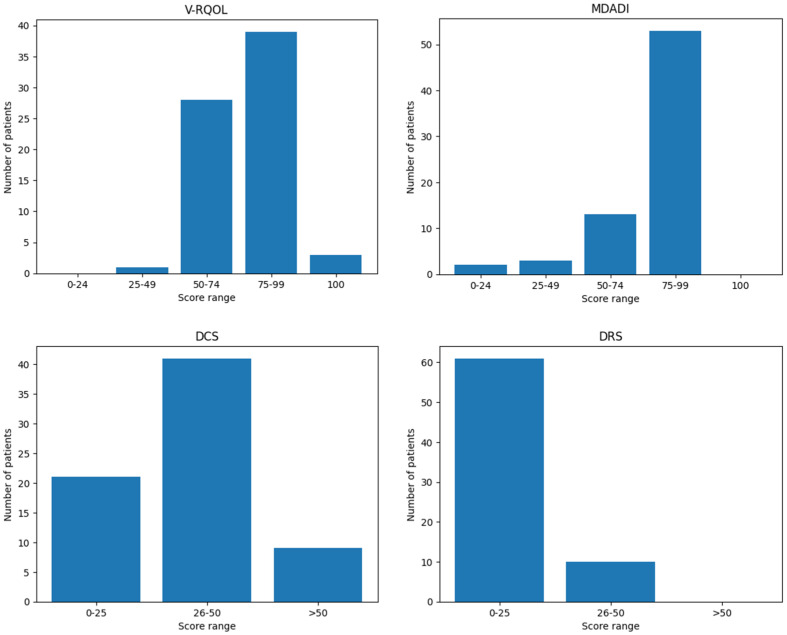
Distribution of patients across score ranges for V-RQOL, MDADI, DCS, and DRS questionnaires.

No statistically significant correlations were observed between V-RQOL scores and patient-related variables such as age, sex, smoking or alcohol habits, or history of prior treatments. Similarly, there were no significant differences in voice-related quality of life based on the type of surgical procedure performed or the need for adjuvant therapy (Figure 2).

### 3.3. MD Anderson Dysphagia Inventory (MDADI)

The mean MDADI score was 78.8 out of 100 (SD ± 17.73), indicating a low overall impact of dysphagia on patients’ quality of life. As with the V-RQOL, higher scores correspond to better perceived swallowing-related quality of life. The distribution of scores by category is summarized in Table 3.

No significant correlations were found between MDADI scores and patient characteristics such as age, sex, smoking or alcohol habits, or previous treatments. Additionally, no statistically significant differences were observed in swallowing-related satisfaction across different types of surgical procedures.

However, a statistically significant difference was found between patients who underwent adjuvant therapy and those who did not (*p* = 0.034). Despite this, linear regression analysis showed that the difference was not independently attributable to adjuvant therapy (*p* = 0.224; adjusted R^2^ = 0.007) (Figure 2).

### 3.4. Decisional Conflict Scale (DCS)

DCS scores reflect the level of difficulty experienced by patients when deciding whether to undergo surgery; higher scores indicate greater decisional uncertainty. In this cohort, the mean DCS score was 34.19 out of 100 (SD ± 12.97). Table 3 presents the distribution of patients according to low, moderate, and high decisional conflict levels.

No significant differences in DCS scores were observed in relation to age, sex, previous treatments, type of surgical procedure, or receipt of adjuvant therapy (Figure 2).

### 3.5. Decisional Regret Scale (DRS)

Similar to the DCS, the Decisional Regret Scale (DRS) evaluates the emotional response to the decision to undergo surgery, with higher scores indicating greater regret. In this cohort, the mean DRS score was 13.0 out of 100 (SD ± 11.20). The distribution of scores across different regret levels is shown in Table 3.

As with the DCS, no significant associations were found between DRS scores and patient-related variables such as age, sex, prior treatments, type of surgery, or receipt of adjuvant therapy (Figure 2).

### 3.6. Brief Pain Inventory

At the time of questionnaire completion, 56 patients (78.87%) reported no pain within the preceding 24 h, while 15 patients (21.13%) reported pain in one or more body regions. Among these 15, the most frequently reported site was the neck (11 patients, 73.33%), followed by the shoulders (6 patients, 40%), back (1 patient, 6.67%), face (1 patient, 6.67%), and ears (1 patient, 6.67%). Of the 15 patients experiencing pain, 6 (40%) had undergone adjuvant therapy.

Regarding pain management, 9 out of 15 patients (60%) were not receiving any treatment. Among those treated, 4 (26.67%) used regular analgesics, and 2 (13.33%) used anti-inflammatory medications. On average, pain relief achieved with treatment was 66.7%.

Table 4 summarizes reported pain intensity (minimum, maximum, average, and current) during the 24 h prior to questionnaire completion.

**Figure 3:**
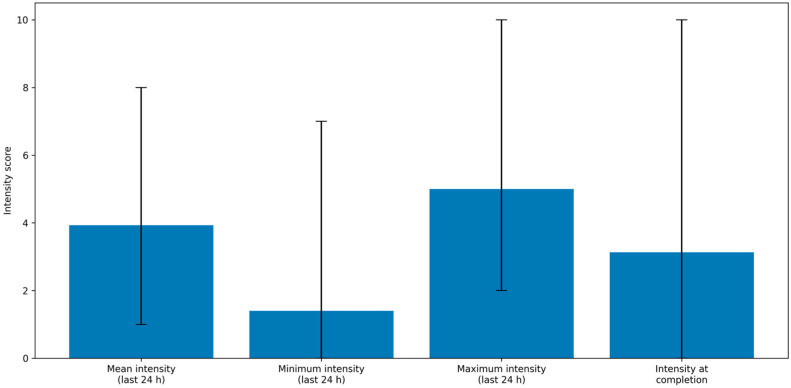
Graphical representation of pain intensity measures. Bars represent mean values, while error bars indicate the minimum and maximum observed values for each variable.

Chronic pain was significantly associated with worse scores on both the V-RQOL and MDADI scales (*p* = 0.01 and *p* < 0.01, respectively), while no significant associations were found with DCS or DRS scores (Table 5). Linear regression analysis showed that chronic pain was associated with an average decrease of 10.34 points in V-RQOL scores (*p* = 0.005; adjusted R^2^ = 0.097) and a 15.67-point reduction in MDADI scores (*p* = 0.002; adjusted R^2^ = 0.12) (Figure 3).

No associations were found between the presence of chronic pain and age, sex, or previous treatments. However, adjuvant therapy was significantly associated with chronic pain (Chi^2^ = 13.57, Cramér’s V = 0.43, *p* < 0.01). Logistic regression confirmed this association, with a Nagelkerke R^2^ of 0.235 and an odds ratio (OR) of 9.524 (*p* = 0.01).

## 4. Discussion

Traditionally, the success of surgical procedures has been assessed using objective, outcome-based parameters, with a primary focus on oncological and functional results. However, there is growing recognition of the importance of incorporating the patient’s perspective, as subjective perceptions of outcomes may differ significantly even in the presence of clinically satisfactory results [20,21]. This underscores the need to identify patients’ perceived priorities before surgery, in order to adopt a more personalized and patient-centered therapeutic approach.

While survival is generally considered the primary concern among patients, those affected by laryngeal cancer may place a high value on preserving voice and swallowing function, even at the expense of survival-related metrics. In this study, 71 patients (58 males and 13 females) subjected to open partial horizontal laryngectomy (OPHL) between 2005 and 2024 in a single Institution were evaluated, with the aim of investigating patient priorities and long-term satisfaction. The Priority Scale developed by Sharp et al. in 1998 [12], a validated instrument designed to guide shared decision-making between patients and clinicians when selecting between treatments with significant trade-offs, such as surgery and radiotherapy, was used.

The most frequently selected patient priorities were “being cured of cancer” (98.6%) and “living as long as possible” (83.1%), followed by “maintaining my natural voice” (33.8%) and “being free of pain” (28.2%). Interestingly, “being able to swallow all foods and liquids” ranked only seventh, while “returning to my usual activities as soon as possible” (52.1%) and “maintaining physical appearance” (62.0%) were among the least prioritized items. These findings suggest that patients undergoing OPHL are aware of the long recovery process and potential changes in appearance. Age was shown to influence patient priorities, emphasizing the need for clinicians to consider age-specific differences during preoperative counseling.

The results of this study are consistent with the conceptual framework proposed by Sharp et al. [12], who first introduced the Priority Scale to support patient involvement in complex treatment choices. Similar to their observations, the findings of the present study confirm that oncological control remains the dominant priority, while functional aspects, although relevant, tend to assume a secondary but individualized role in the decision process. More recent studies [22,23] applying the Priority Scale in head and neck oncology have also highlighted its value in structuring shared decision-making discussions, helping clinicians tailor counseling to patients’ personal values and expectations, which is consistent with the age-related variability observed in our cohort.

The top-ranked priorities were further explored through validated questionnaires. With a mean follow-up of 52.4 months, disease-free survival rates were 97.2% at 5 years, 93% at 10 years, and 90.2% at 15 years, confirming the long-term oncological effectiveness of OPHL.

Swallowing recovery after OPHL, historically considered the most challenging functional outcome, appears to be better than expected. Although the immediate postoperative impact on swallowing is significant, most patients regain satisfactory long-term function. Only a minority (10–20%) experience chronic aspiration, typically with low risk of aspiration pneumonia. The MD Anderson Dysphagia Inventory (MDADI) showed a mean score of 78.8/100, indicating a limited impact of dysphagia on quality of life. No significant differences were observed between surgical subtypes, except for patients receiving adjuvant therapy (*p* = 0.034); however, this was not confirmed in linear regression analysis (*p* = 0.224), suggesting that other factors may influence swallowing-related outcomes.

These findings are consistent with the literature. A systematic review by Schindler et al. reported MDADI scores between 78 and 92 following OPHL type II. Comparative studies also showed no significant functional differences—including MDADI scores—between type IIa and IIIa OPHLs [24]. Similarly, after type III OPHL, Schindler et al. reported a mean MDADI score of 78.5, confirming minimal impact of resection extent on swallowing-related quality of life [25]. However, Pizzorni et al. highlighted a weak correlation between MDADI scores and actual dysphagia severity, suggesting that patients may underreport functional impairment. Therefore, instrumental swallowing evaluation remains essential in postoperative assessment [26].

Voice quality, historically viewed as less critical than swallowing function, is now recognized as a more variable and less predictable functional outcome after OPHL. Despite this, the mean Voice-Related Quality of Life (V-RQOL) score was 77.4/100, indicating overall patient satisfaction with vocal outcomes. No significant correlations were found with age, sex, lifestyle factors, surgical approach, or adjuvant therapy, suggesting that current techniques provide an acceptable level of voice quality for most patients. While electroacoustic and aerodynamic assessments often reveal severe voice impairment after OPHL type II or III, patients typically report only moderate impact on physical, emotional, and functional domains, highlighting a possible discrepancy between clinical judgment and patient perception.

Pain management also emerged as an important aspect of postoperative quality of life. Chronic pain was reported by 21% of patients, with a mean intensity of 3.93/10 in the previous 24 h. However, only 21% of these patients required analgesic therapy. Chronic pain was significantly associated with worse scores on both the V-RQOL and MDADI (*p* < 0.01), emphasizing the need for effective pain control to optimize quality of life.

The Decisional Conflict Scale (DCS) indicated a mean score of 34.19/100, suggesting a low-to-moderate level of decisional uncertainty. No significant differences were found based on age, sex, prior treatments, surgical type, or adjuvant therapy. While this is encouraging, it highlights room for improvement in preoperative communication. Enhanced dialogue and psychological support could further reduce patient uncertainty and facilitate truly informed decision-making.

The Decisional Regret Scale (DRS) revealed a mean score of 13/100, indicating a very low level of regret. This suggests that, despite the complexity of OPHL, outcomes generally align with patients’ expectations and contribute to a positive retrospective perception of treatment. However, this finding should be interpreted with caution, as potential sources of bias—particularly response bias and survivor bias—may have influenced the results. Only patients who were alive, disease-free, and compliant with follow-up completed the questionnaire, potentially skewing responses toward more favorable perceptions.

These results are in line with recent evidence reported in the literature. A large systematic review and meta-analysis published in 2024 in JAMA Otolaryngology–Head and Neck Surgery [27] analyzed decisional conflict and decisional regret in head and neck oncology and demonstrated that decisional regret is generally low across different treatment modalities, particularly in long-term survivors, whereas decisional conflict tends to decrease over time as patients retrospectively reassess their treatment choice in light of oncological outcomes. Importantly, it is also highlighted that lower decisional regret is frequently associated with favorable oncologic control, prolonged survival, and adaptation to functional sequelae, whereas higher regret is more often reported in patients experiencing recurrence or major complications. These findings support the interpretation that, in successfully treated patients, treatment choices are retrospectively perceived as appropriate and justified, even in the presence of relevant functional limitations.

Consistently with these observations, the present study reports low decisional conflict and regret following OPHL, reinforcing the hypothesis that, when treatment achieves durable disease control and acceptable long-term function, patient expectations are largely met and retrospective satisfaction remains high. However, the JAMA meta-analysis also underlined that decisional outcomes may be influenced by methodological biases, including survivor bias and response bias, particularly in studies assessing only long-term survivors. This is highly relevant to the cohort of the present, in which only disease-free patients compliant with long-term follow-up were evaluated. Therefore, while the findings of the present study fit within the broader international evidence and support the role of OPHL as a treatment associated with high acceptance and limited decisional regret, they should nonetheless be interpreted cautiously within this methodological framework.

Nevertheless, this study has some limitations. Being conducted in a single tertiary referral center, its findings may not be fully generalizable. The relatively small sample size may limit the statistical power of the analysis and increase the risk of selection bias, reducing the robustness of some conclusions. Data were collected from patients who were disease-free at a distance from treatment, which may have positively influenced responses. Additionally, as data collection occurred within the surgical center, there is potential for response bias, with patients possibly hesitant to express dissatisfaction regarding a life-saving procedure

Overall, the findings underscore the importance of a multidimensional approach when evaluating outcomes after OPHL. While survival remains the primary concern, factors such as voice quality, swallowing function, and pain management play critical roles in patient-perceived quality of life. Age and individual preferences should be carefully considered during preoperative counseling to ensure personalized care.

Finally, the low levels of decisional conflict and regret indicate that the current preoperative process is largely effective. However, further improvements in patient–clinician communication—such as allocating more time for discussions and incorporating psychological support tools—could help reduce decisional uncertainty and enhance overall satisfaction. This study reinforces the value of patient-centered care and tailored treatment strategies in optimizing both clinical outcomes and patient experience following OPHL.

## 5. Conclusions

This study provides valuable insights into the priorities of patients undergoing open partial horizontal laryngectomy (OPHL) and their perception of postoperative quality of life. The findings highlight the need for a therapeutic approach that goes beyond traditional oncological and functional outcomes, fully incorporating the patient’s perspective. While survival remains a central objective, other factors—such as voice quality, swallowing function, and pain management—play a crucial role in overall patient satisfaction.

The study’s limitations, including its single-institution design and the potential for response bias due to data collection within the treating center, underscore the importance of future multicenter studies to validate and expand upon these findings. In addition, longer follow-up periods may provide more detailed information on how patients’ perceptions evolve over time. Monitoring patient-reported outcomes during follow-up could help tailor rehabilitation strategies—such as injection laryngoplasties—to better meet individual needs.

It is hoped that this approach may contribute to identify, even in the preoperative phase, patients at higher risk of suboptimal functional and psychological outcomes, thereby enabling the early implementation of targeted rehabilitative and psychological interventions.

In summary, integrating patient priorities and preferences into the preoperative decision-making process is essential to optimize both clinical outcomes and quality of life. This patient-centered approach represents a critical step toward ensuring that treatment is not only medically effective but also aligned with the personal expectations and values of each patient.

## Figures and Tables

**Table 1:** Demographic data.

			No. of Patients (%)
Age	Mean ± SD		65.68 ± 9.42
	Range		40–85
Age at surgery	Mean ± SD		60.66 ± 8.74
	Range		37–76
Sex	Male		58 (81.69)
	Female		13 (18.31)
Smoking	No		15 (21.13)
	Yes		56 (78.87)
Alcohol	No		64 (90.15)
	Yes		7 (9.86)
Previous treatment	No		58 (81.69)
	Yes		13 (18.31)
		Type I cordectomy	2 (15.38)
		Type II cordectomy	4 (30.77)
		Type III cordectomy	2 (15.38)
		Type IV cordectomy	1 (7.69)
		Type V cordectomy	4 (30.77)
cT	2		28 (39.44)
	3		42 (59.15)
	4		1 (1.41)
cN	0		66 (92.96)
	1		5 (7.04)
pT	2		32 (45.07)
	3		31 (43.66)
	4		7 (9.86)
pN	0		53 (74.65)
	1		7 (9.86)
	2b		1 (1.41)
	2c		2 (2.82)
	3a		1 (1.41)
	3b		1 (1.41)
OPHL type	I		8 (11.27)
	I + BOT		2 (2.82)
	IIA		10 (14.08)
	IIA + ARY		34 (47.89)
	IIB		2 (2.82)
	IIB + ARY		4 (5.63)
	IIIA		4 (5.63)
	IIIA + CAU		6 (8.45)
	IIIB + CAU		1 (1.41)
ND type	none		6 (8.45)
	ipsilateral		45 (63.38)
	bilateral		20 (28.17)
Adjuvant treatment	none		57 (80.28)
	RT		7 (9.86)
	CRT		6 (8.45)
	radioiodine		1 (1.41)

BOT: base of tongue, ARY: arytenoid, CAU: crico-arytenoid unit. For further details on the different types of OPHL and the surgical techniques, please refer to Succo et al. [11].

**Table 2:** Priority Scale: differences in priorities indicated by young patients (≤ 65 years) compared to non-young patients (>65 years).

	Mean (≤65 Years)	Mean (>65 Years)	*p* Value
Keeping my natural voice	5.71	4.78	0106
Being able to chew normally	6.58	5.95	0.207
Being able to swallow all foods and drinks	6.00	6.18	0.856
Being cured of my cancer	1.42	1.45	0.907
Having no pain	8.06	7.70	0.537
Keeping my appearance unchanged	10.16	9.28	0.073
Returning to my activities as soon as possible	7.87	9.50	0.050
Having a normal amount of energy for me	6.61	6.73	0.815
Keeping my normal sense of taste and smell	7.61	7.70	0.958
Having my speech understood easily	5.29	5.58	0.404
Living as long as possible	2.29	2.90	0.781
Having a comfortably moist mouth	10.39	10.23	0.543

**Table 3:** Results of V-RQOL, MDADI, DCS, DRS questionnaires.

	Points/100	No. of Patients (%)
V-RQOL	0–24	0 (0)
	25–49	1 (1.41)
	50–74	28 (39.44)
	75–99	39 (54.93)
	100	3 (4.23)
MDADI	0–24	2 (2.82)
	25–49	3 (4.23)
	50–74	13 (18.31)
	75–99	53 (74.65)
	100	0 (0)
DCS	0–25	21 (29.58)
	26–50	41 (57.75)
	>50	9 (12.68)
DRS	0–25	61 (85.92)
	26–50	10 (14.08)
	>50	0 (0)

**Table 4:** BPI results.

	Mean	Min	Max
Mean intensity in the last 24 h	3.93	1	8
Minimum intensity in the last 24 h	1.4	0	7
Maximum intensity in the last 24 h	5	2	10
Intensity at the time of completion	3.13	0	10

**Table 5:** Association of chronic pain and V-RQOL, MDADI, DCS, DRS.

	Mean (Patients with Pain)	Mean (Patients Without Pain)	*p* Value
V-RQOL	69.28	79.63	0.010
MDADI	66.40	82.07	0.000
DCS	39.08	32.88	0.086
DRS	12.67	13.04	0.769

## Data Availability

The authors clarify that, as this study is a retrospective series spanning 20 years and was conducted in different hospitals, data sharing outside the individual institutions is not possible, because explicit consent for such sharing was not obtained from all patients.
